# Cancer Cell Growth Inhibitory Effect of Bee Venom via Increase of Death Receptor 3 Expression and Inactivation of NF-kappa B in NSCLC Cells

**DOI:** 10.3390/toxins6082210

**Published:** 2014-07-25

**Authors:** Kyung Eun Choi, Chul Ju Hwang, Sun Mi Gu, Mi Hee Park, Joo Hwan Kim, Joo Ho Park, Young Jin Ahn, Ji Young Kim, Min Jong Song, Ho Sueb Song, Sang-Bae Han, Jin Tae Hong

**Affiliations:** 1 College of Pharmacy and Medical Research Center, Chungbuk National University, 52 Naesudong-ro, Heungdeok-gu, Cheongju, Chungbuk 361-763, Korea; E-Mails: cge7777@naver.com (K.E.C.); hcj0629@naver.com (C.J.H.); g09010327@nate.com (S.M.G.); pmh5205@hanmail.net (M.H.P.); kjh074@korea.kr (J.H.K.); jhp31888@naver.com (J.H.P.); ayjinya@naver.com (Y.J.A.); ikiru21@naver.com (J.Y.K.); shan@chungbuk.ac.kr (S.-B.H.); 2 Department of Obstetrics and Gynecology, Daejeon St. Mary’s Hospital, College of Medicine, the Catholic University of Korea, 64 Daeheung-ro, Jung-gu, Daejeon 301-723, Korea; E-Mail: bitsugar@hanmail.net; 3 College of Oriental Medicine, Gachon University, San 65, Bokjeong-dong, Sujeong-gu, Seongnam, Gyeonggii 461-701, Korea; E-Mail: hssong70@gachon.ac.kr

**Keywords:** bee venom, apoptosis, death receptors, NF-κB

## Abstract

Our previous findings have demonstrated that bee venom (BV) has anti-cancer activity in several cancer cells. However, the effects of BV on lung cancer cell growth have not been reported. Cell viability was determined with trypan blue uptake, soft agar formation as well as DAPI and TUNEL assay. Cell death related protein expression was determined with Western blotting. An EMSA was used for nuclear factor kappaB (NF-κB) activity assay. BV (1–5 μg/mL) inhibited growth of lung cancer cells by induction of apoptosis in a dose dependent manner in lung cancer cell lines A549 and NCI-H460. Consistent with apoptotic cell death, expression of DR3 and DR6 was significantly increased. However, deletion of DRs by small interfering RNA significantly reversed BV induced cell growth inhibitory effects. Expression of pro-apoptotic proteins (caspase-3 and Bax) was concomitantly increased, but the NF-κB activity and expression of Bcl-2 were inhibited. A combination treatment of tumor necrosis factor (TNF)-like weak inducer of apoptosis, TNF-related apoptosis-inducing ligand, docetaxel and cisplatin, with BV synergistically inhibited both A549 and NCI-H460 lung cancer cell growth with further down regulation of NF-κB activity. These results show that BV induces apoptotic cell death in lung cancer cells through the enhancement of DR3 expression and inhibition of NF-κB pathway.

## 1. Introduction

In a recent study, lung cancer was expected to account for 26% of all female cancer deaths and 29% of all male cancer deaths in 2012 [1]. Lung cancer is very difficult to discover in the initial stage because it often does not cause any symptoms until it has spread to others organs [2,3]. Although several anticancer agents have been used to treat lung cancer, chemo-resistance is a major obstacle hindering the successful treatment of lung cancer patients [4]. Thus, appropriate chemopreventive compounds reducing or overcoming resistance is a very hopeful strategy for chemotherapy of lung cancer [5].

Bee venom (BV) has been traditionally used for the treatment of back pain, rheumatism, and skin diseases due to its antibacterial, antiviral, and anti-inflammatory effects [6]. Previously, our studies have shown that BV inhibits cancer cell growth through the induction of apoptosis of prostate [7] and ovarian cancer cells [8,9]. BV treatment induces both caspase-dependent and caspase-independent apoptotic cell death through the activation of intracellular Ca(2+)-modulated intrinsic death pathway in human bladder cancer cells [10]. However, there is no information about the effect of BV on death receptors (DRs) mediated apoptosis in human lung cancer cells.

Apoptosis plays an important role in anti-cancer effects of chemotherapeutics. Stimulation of DR expression is implicated in the induction of apoptosis in cancer cells, especially chemoresistant cancer cells. DRs are activated by the interaction of DRs with their ligands (interaction of DR1 with tumor necrosis factor (TNF); DR2 with Fas ligand (FasL); DR3 with Apo3L/TWEAK; DR4 and DR5 with TNF-related apoptosis-inducing ligand (TRAIL); ligand of DR6) has not been exactly defined [11]. When DRs bind to their ligands, death domains recruit the intracellular adaptor protein FADD (Fas-associated death domain protein) which results in the activation of caspases, including caspases-3, -8 and -9, as well as an increase of Bax and decrease of BCl-2 to induce apoptosis [12,13,14].

The nuclear factor kappa B (NF-κB) family plays an important role in several human cancer cell growths [15,16,17]. NF-κB gene products have also been shown to have importance in proliferative and anti-apoptotic activities that could contribute to the tumor development, progression, and resistance to therapy of tumor cells [17]. In lung cancer cells, a clear correlation between inactivation of NF-κB and an enhanced therapeutic effect was observed [18,19,20]; Genistein enhanced antitumor effects due to greater reduction in the DNA-binding activity of NF-κB [19]. Some chemotherapeutic agents such as cisplatin, docetaxel, and doxorubicin induced the activation of NF-κB in the resistance of several cancer cells, and the reduction of NF-κB has been believed to be responsible in the killing of drug resistant cancer cells [7,21,22]. Inactivation of NF-κB in combination with chemotherapeutic agents also leads to better tumor cell killing effects in human cancers [23]. Thus, agents capable of suppressing NF-κB pathway may be potentially useful in the prevention and management of lung cancer growth and resistance.

## 2. Results

### 2.1. Effect of BV on Cell Growth in Lung Cancer Cells

**Figure 1:**
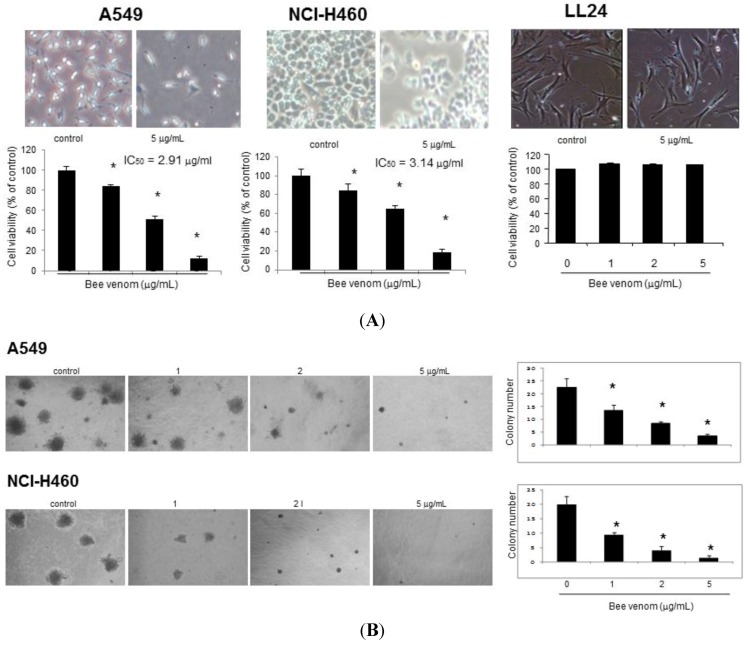
Effect of BV on cell viability and colony formation of lung cancer cells. (**A**) Concentration-dependent effect of bee venom (BV) on cell viability of A549 and NCI-H460 cells as well as normal lung cell LL24. Relative cell survival rate was determined by counting live and dead cells. Results were expressed as a percentage of viable cells; (**B**) Effect of BV-induced inhibition of colony formation in A549 and NCI-H460 cells. Data are expressed as the mean ± S.D. of three experiments. * *p* < 0.05 indicates statistically significant differences from control group.

To assess the inhibitory effect of BV on cell growth of A549 and NCI-H460 cells, we analyzed cell viability by cell counting. Cells were treated with several concentrations of BV (1, 2 and 5 μg/mL). As shown in Figure 1A, BV inhibited cell proliferation of lung cancer cells in a concentration-dependent manner. Morphologic observation showed that cells were gradually reduced in size and changed into a small round single cell shape by the treatment of BV on lung cancer cells. A 48 hour treatment of BV inhibited A549 cell growth with IC_50_ value of 2 μg/mL and NCI-H460 cell growth with IC_50_ value of 3 μg/mL, respectively. However, the normal lung cell (LL24 cells) growth was not affected by BV. To further demonstrate the cancer cell growth inhibitory effect of BV, we assayed the colony forming assay. The number of colonies was concentration-dependently decreased by BV (Figure 1B), and the concentration of BV for inhibition of colony formation was similar to the concentration for cell growth inhibition. Thus, BV inhibits lung cancer cell growth.

### 2.2. Apoptotic Cell Death by BV

**Figure 2:**
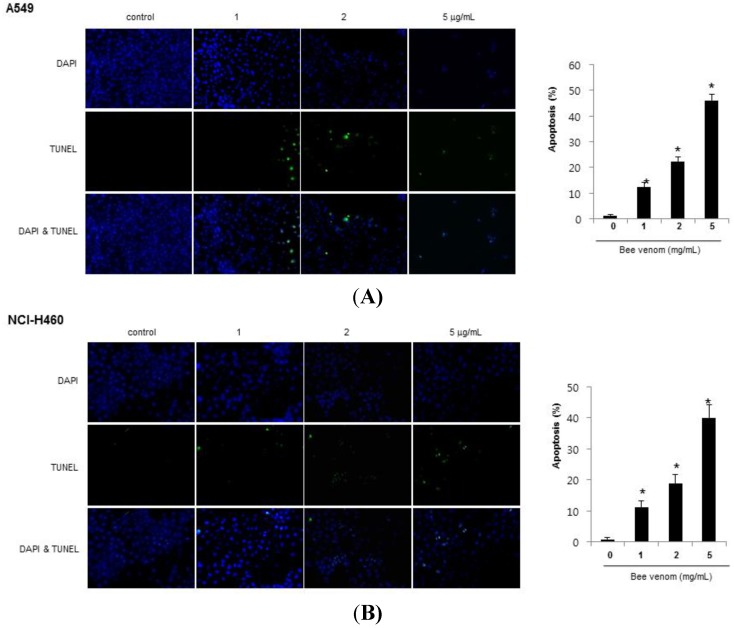
Effect of BV on apoptotic cell death. Lung cancer cells were treated with BV (1, 2 and 5 μg/mL) for 24 h, and then labeled with DAPI and TUNEL solution. Total number of cells in a given area was determined by using DAPI nuclear staining (fluorescent microscope). A green color in the fixed cells marks TUNEL-labeled cells. Apoptotic index was determined as the DAPI-stained TUNEL-positive cell number/total DAPI stained cell number × 100 (magnification, 200×). Data are expressed as the mean ± S.D. of three experiments. * *p* < 0.05 indicates statistically significant differences from control cells. (**A**) Apoptotic cell death of A549; (**B**) Apoptotic cell death of NCIH460.

To determine whether the inhibition of cell growth by BV was due to the induction of apoptotic cell death, we evaluated the changes in the chromatin morphology of cells by using DAPI staining followed by TUNEL staining assays, and then the double labeled cells were analyzed by a fluorescence microscope. The IC_50_ with cell growth inhibition, DAPI-stained TUNEL-positive cells were significantly increased by BV (1–5 μg/mL) in both A549 and NCI-H460 cells in a concentration-dependent manner (Figure 2).

### 2.3. Expression of Apoptotic Regulatory Proteins and Death Receptor by BV

**Figure 3:**
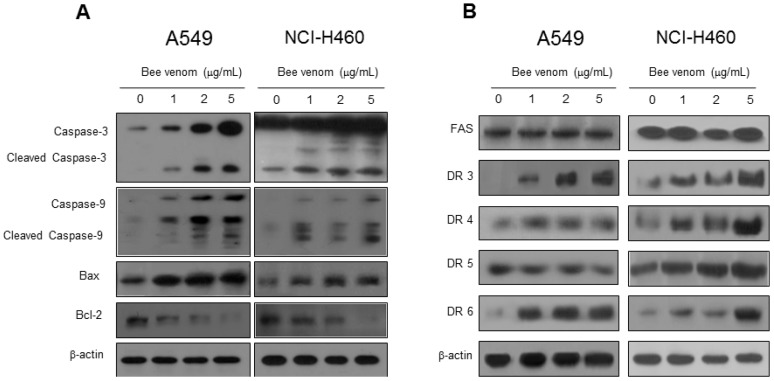
Effect of BV on the expression of apoptosis regulatory proteins. (**A**) Expression of apoptosis regulatory proteins related intrinsic pathway was determined using Western blot analysis with antibodies against caspase-3, caspase-9, bax, bcl-2 and β-actin (internal control); (**B**) Extrinsic pathway was determined using Western blot analysis with antibodies against FAS, DR3, DR4, DR5, DR6 and β-actin (internal control). Each band is representative for three experiments.

**Figure 4:**
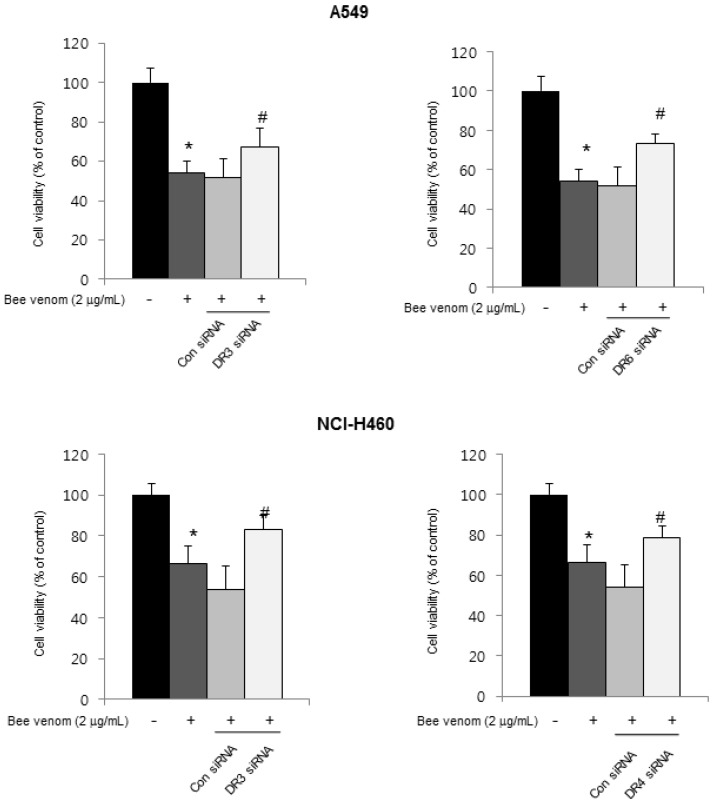
Effect of DR knockdown on BV-induced lung cancer cells growth. Lung cancer cells were transfected with non-targeting control siRNA, DR3 or DR4 siRNA (100 nM) for 24 h; then, treated with BV (2 μg/mL) at 37 °C for another 24 h. Relative cell survival rate was determined by counting live and dead cells. Results were expressed as a percentage of viable cells. Data are expressed as the mean ± S.D. of three experiments. * *p* < 0.05 indicates statistically significant differences from control cells. ^#^ *p* < 0.05 indicates significantly different from BV treated cells.

To figure out the mechanisms of apoptotic cell death, expression of apoptotic cell death related proteins was investigated by Western blots. The expressions of apoptotic proteins (cleaved-caspases 3, cleaved-caspases 9 and Bax) were increased, but Bcl-2 was decreased in both A549 and NCI-H460 cells (Figure 3A). Apoptosis also can be induced by the stimulation of DRs’ expression. Therefore, to investigate the expression of DRs in cancer cells undergoing apoptotic cell death, the expression of death receptor proteins such as DR3 and DR6 in A549 cells and DR3, DR4 and DR6 in NCI-H460 cells were increased (Figure 3B). To further investigate the involvement of DR expression in cell death, cells were transfected with 100 nM siRNA of DRs for 24 h. Cell growth was assessed after the treatment with BV (2 μg/mL) for 24 h. As shown in Figure 4, the transfection of DR3 and DR6 siRNA reversed BV-induced cell growth inhibition in A549 cells, and DR3 and DR4 siRNA also reversed BV-induced cell growth inhibition in NCI-H460 cells (Figure 4).

### 2.4. Involvement of NF-κB Signaling Pathway in Apoptotic Cell Death by BV

**Figure 5:**
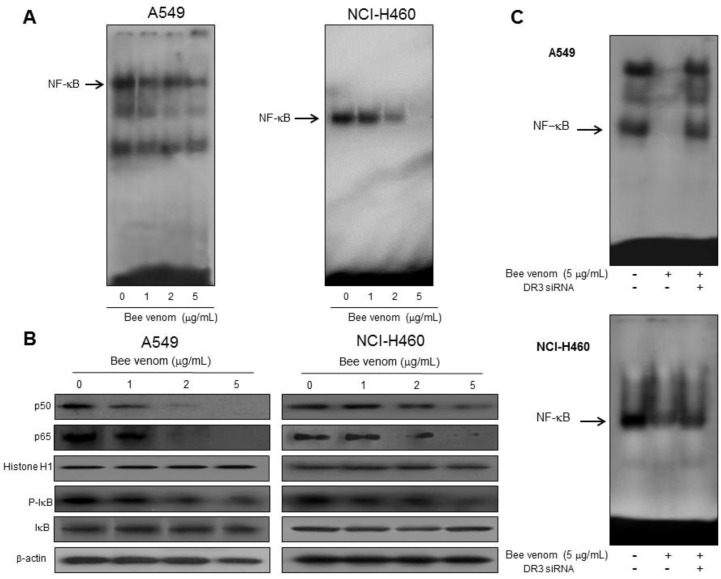
Effect of BV on NF-κB activation in lung cancer cells. Lung cancer cells treated with BV (1, 2 and 5 μg/mL) for 1 h, and then were lysed. (**A**) Nuclear extract was incubated in binding reactions of ^32^P-end-labeled oligonucleotide containing the κB sequence. The present EMSA results are representatives of three experiments; (**B**) Cytosolic proteins were used to determine expression of IκB, p-IκB and β-actin (internal control) and nuclear proteins were used to expression of p50, p65 and histone H1 (internal control) in lung cancer cells. Each band is representative for three experiments; (**C**) Effect of siRNA of DR3 on the BV-induced NF-κB inactivation. Each band is representative of three experiments.

A decrease in activity of NF-κB has been shown to be involved in apoptotic cell death in many cancer cells. Hence, we examined the DNA binding activity of NF-κB with EMSA (Figure 4A). BV has been shown to negatively regulate NF-κB by means of protein–protein interaction [6]. NF-κB activation in cancer cells highly correlates with the resistance to apoptotic cell death [24]. Therefore, to investigate whether BV can inactivate NF-κB, and thereby hinder its anti-apoptotic ability ultimately causing the cells to undergo apoptotic cell death, we assessed NF-κB activity in lung cancer cells treated with various concentrations of BV for 1 h. In Figure 5, the constitutive activation of NF-κB was reduced by BV in a concentration dependent manner. To interpret the EMSA results, we performed Western blotting for the NF-κB proteins. Along with the inhibitory effect on NF-κB DNA binding activity (Figure 5A), we also found that BV concentration-dependently and markedly inhibited phosphorylation of IκB and translocation of p50 and p65 in both A549 and NCI-H460 cells (Figure 5B). Furthermore, as shown in Applendix, confocal microscopy observation showed significant inhibition of p50 and p65 nuclear translocation by BV treatment. To see whether DR3 expression could be related with NF-κB activity, we employed siRNA of DR3 for NF-κB activity. siRNA of DR3 reversed BV-induced NF-κB inactivation in both A549 and NCI-H460 lung cancer cells (Figure 5).

### 2.5. Combination of BV with TWEAK Further Induced DR3 Overexpression and Inactivation NF-κB

**Figure 6:**
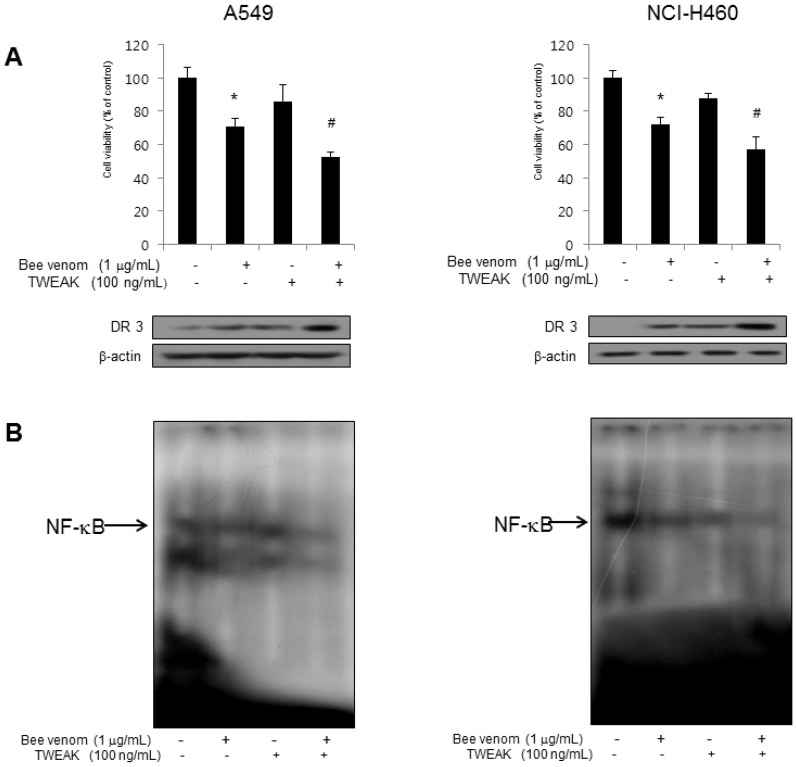
Effect of co-treatment of BV and TWEAK in lung cancer cells. (**A**) Effect of pretreatment with TWEAK on the BV-induced inhibition of lung cancer cells. Lung cancer cells were treated with TWEAK for 24 h, and then treated with BV (1 μg/mL) at 37 °C for another 24 h. Relative cell survival rate was determined by counting live and dead cells. Results were expressed as a percentage of viable cells. The expression of DR3 was determined by Western blot analysis with the antibody against DR3 and β-actin (internal control). Each band is representative for three experiments; (**B**) Effect of TWEAK on the BV-induced NF-κB inactivation. The data are expressed as the mean ± S.D. of three experiments. * *p* < 0.05 indicates statistically significant differences from control cells. ^#^ *p* < 0.05 indicates significantly different from BV treated cells.

Resistance of cancer cells for chemotherapeutics is associated with lower expression of DRs and higher activity of NF-κB. To further study whether BV could overcome chemoresistance of cancer cells, we co-treated lung cancer cells with a combination of a lower concentration of BV (1 μg/mL) and TWEAK, a ligand of DR3, since DR3 was overexpressed by BV and siRNA of DR3 reversed the cell growth inhibitory effect of BV in both cells. After co-treatment, lung cancer cell growth, overexpression of DR3 and inactivation of NF-κB were tested. As shown in Figure 6, we found that co-treatment of BV and TWEAK more inhibited lung cancer cell growth. The expression of DR3 was also significantly higher in the combination treatment of BV and TWEAK compared to the expression by BV or TWEAK treatments alone (Figure 6A). We also found that co-treatment of BV and TWEAK inhibited activation of NF-κB at a higher level compared to BV or TWEAK alone (Figure 6B).

### 2.6. Combinations of BV with Chemotherapeutics on Inhibition of Lung Cancer Cell Growth, DR3 Overexpression and Inactivation of NF-κB

**Figure 7:**
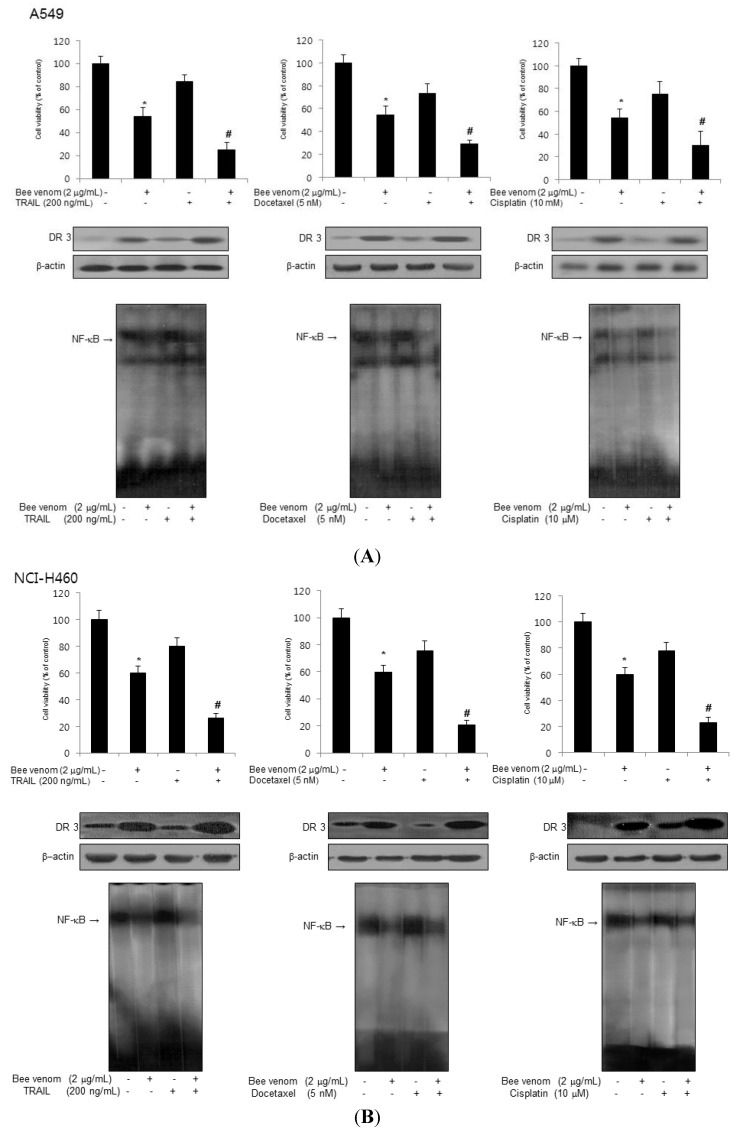
Effect of combinations of BV with chemotherapeutics. (**A**) A549 cells were seeded and treated with the indicated concentrations of BV, TRAIL (or docetaxel or cisplatin) and BV with TRAIL (or docetaxel or cisplatin) combination, and then confirmed cell viability, expression of DR3 and activation of NF-κB; (**B**) NCI-H460 cells were seeded and treated with the indicated concentrations of BV, TRAIL (or docetaxel or cisplatin) or BV with TRAIL (or docetaxel or cisplatin) combination, and then confirm cell viability, expression of DR3 and activation of NF-κB. Data are expressed as the mean ± S.D. of three experiments. * *p* < 0.05 indicates statistically significant differences from the control group, and ^#^ *p* < 0.05 indicates statistically significant differences from BV or TRAIL (or docetaxel or cisplatin) treated group.

We treated lung cancer cells with 2 μg/mL BV with 200 ng/mL TRAIL, 5 nM docetaxel or 10 μM cisplatin since we previously found the cells were resistant to these doses of chemotherapeutics [25,26,27]. Treatment of 2 μg/mL BV in combination with 200 ng/mL TRAIL, 5 nM docetaxel or 10 μM cisplatin resulted in a strong synergistic inhibitory effect on cell growth through further overexpression of DR3 and inactivation of NF-κB in A549 cells (Figure 7A) and in NCI-H460 cells (Figure 7B). These results suggest that the combination of BV with lower doses of chemotherapeutics elicited significantly greater inhibition of lung cancer cell growth compared with either agent alone through further overexpression of DR3 and inactivation of NF-κB.

## 3. Discussion

Bee venom (BV) has been used for the treatment of various diseases (back pain, rheumatism and skin diseases) and inhibits cancer cell growth through the induction of apoptosis in several types of cancer cells. The IC_50_ of cell growth inhibition were 1.5~2.9 μg/mL in prostate cancer cells, and 1.5 μg/mL in ovarian cancer cells, and about 7 μg/mL in human bladder cancer cells, respectively. Similar to these data, our present data also showed that BV inhibited cell growth of lung cancer cells in a concentration-dependent manner, and 48 h treatment of BV inhibited A549 cell growth with IC_50_ value of 2 μg/mL, and NCI-H460 cell growth with IC_50_ values of 3 μg/mL, respectively. However, the normal lung cell growth was not changed by BV. This data indicates that BV could be effective in lung cancer cell growth inhibition with a similar range of concentration. However, a clear mechanism is not identified. The possible molecular targets of BV and its components for inhibition of cancer cell growth are numerous and different [28]. Previous studies have shown that BV and its components induce apoptosis via activation of caspase [7,8,29,30], phospholipases A2 (PLA2) [31], matrix metalloproteinase-2 (MMP-2) [32] and VEGF [28,33]. In this study, we demonstrated that BV inhibited cancer cell growth in NSCLC (non-small cell lung cancer) A549 and NCI-H460 cells through the induction of apoptosis via increase of DR expression and inhibition of NF-κB pathway.

Several studies have demonstrated that natural compound-induced apoptosis in cancer cells could be related with the increase of DR expression. Indomethacin and sulindac sulfide, one of the major metabolites of sulindac, activate caspase 8 and induce apoptosis by a fas-associating protein with death domain (FADD)-dependent mechanism in Jurkat T cells [13]. Sulindac sulfide is believed to mediate its antitumorigenic effects by inducing apoptosis through an up-regulated DR5 and activated caspase 8 in colon and prostate cancer cell lines [14]. Our results showed that the expression of DR such as DR3 and DR6 in A549 lung cancer cell and DR3, DR4 and DR6 in NCI-H460 lung cancer cell were increased. Moreover, treatment of DR3 and DR6 siRNA in A549, and DR3 and DR4 siRNA in NCI-H460 reversed BV-induced lung cancer cell growth inhibition. Especially in both lung cancer cells, DR3 siRNA reversed BV-induced cell growth inhibition. Our present data also showed that with the co-treatment of BV with TWEAK, the DR3 ligand more effectively inhibited lung cancer cell growth. These results indicate that increased expression of DR3 could be significant in BV-induced lung cancer cell growth inhibition although differential DRs could be involved depending on the cell type. DR ligands such as TRAIL induce apoptosis in a wide range of malignant cells, but several cancers show resistance to TRAIL. However, an increase of the DR expression could overcome human cancer resistance to TRAIL [9]. TRAIL has the ability of a combination effect with other anticancer agents by increasing DR expression. For example, co-treatment of TRAIL with bortezomib increases anti-cancer effects through the overexpression of DRs in mammary carcinoma cells [34]. The combination of TRAIL with gemcitabine also synergistically increases anti-cancer effects through the expression of DRs in urothelial carcinoma cells [35]. Favokawain B (FKB) exerts a synergistic apoptotic effect when combined with TRAIL by increasing the expression of DR5 in prostate cancer [36]. Ozarelix-resistant cancer cells can be sensitized to TRAIL by co-treatment [37]. In our results, low effects of lung cancer cell growth inhibition by TRAIL, docetaxel and cisplatin were observed, but when these compounds were co-treated with BV, a noticeable increase of lung cancer cell growth inhibition was found. We also confirmed that an ability to overcome chemo-resistance through a co-treatment of these compounds with BV was associated with the increase of DR3 overexpression. In addition, the combination treatment of BV and DR3 ligand TWEAK further inhibited lung cancer cell growth and DR3 expression. It was reported that induction of DR3 was observed to overcome resistance to apoptosis in human erythroleukemic TF-1 cells [38]. Therefore, the ability of BV to overexpress DR3 could be effective for lung cancer cell growth inhibition and overcome chemo-resistance in lung cancer cells.

A cancer cell growth inhibitory effect was correlated with the down-regulation of various cell proliferative genes regulated by NF-κB [39]. Several studies have shown that NF-κB is constitutively activated in lung cancers [10,18,23]. Brucine improved apoptosis of lung cancer cells by inhibiting the activity of NF-κB [40]. Gemcitabine also inhibits lung cancer cells growth through a decrease of NF-κB activity [41] and an acacetin treatment to lung cancer cells also leads to a concentration-dependent inhibition of DNA binding abilities of NF-κB causing cell growth inhibition [42]. In agreement with this notion, our data demonstrated that BV inhibits lung cancer cell growth through the inhibition of DNA binding activity of NF-κB. A decrease of NF-κB DNA binding activity was associated with the inhibitory effect of BV on the IκB phosphorylation and nuclear translocation of p50 and p65 in A549 and NCI-H460 cells. Death signaling may be antagonized by anti-apoptotic modulator proteins such as FLIP, members of the IAP family, or proteins of the Bcl-2 family [43]. BV also repressed the expression of anti-apoptotic proteins (Bcl-2), whereas it increased the expression of pro-apoptotic proteins (Bax, cleaved caspase-3, and cleaved caspase-9) which are regulated by NF-κB. Thus, BV may induce an alteration of apoptosis and anti-apoptosis regulatory protein expression that provides a favorable circumstance of the cancer cells to go into a death status. In addition, when TRAIL, docetaxel and cisplatin were co-treated with BV, a noticeable increase of lung cancer cell growth inhibition was associated with more inactivation of NF-κB than with treatment of these compounds alone. It was found that in chemo-resistant human pancreatic cancer AsPC-1, cell growth was inhibited through the up-regulation of DR3 and down-regulation of NF-κB signaling [44]. We also found that (E)-2, 4-bis (p-hydroxyphenyl)-2-butenal has an inhibitory effect on the growth of lung cancer cells due to the induction of apoptosis through the up-regulation of DRs (DR3, DR5 and DR6) and equally the inactivation of NF-κB [45]. Recent studies on the signaling mechanisms of the DR have revealed that members of the NF-κB and caspase families are key regulators of cell death. TNFAIP3 and NFκBIA, which is found to be up-regulated by TRAIL, are inhibitors of the NF-κB pathway [46,47]. Anti-cancer activity of a novel parthenin analog is associated with the inactivation of NF-κB and up regulation of DR5 in human leukemia HL-60 cells [48]. Thus, it may suggest that the inactivation of NF-κB correlates with the increased expression of DRs. We also founded that treatment of BV with DR3 siRNA reversed inactivation of NF-κB and cell growth inhibitory effects of BV, and also showed that co-treatment of BV with TWEAK has more effectively inactivated NF-κB through overexpression of DR3 in the chemo-resistant status. Thus, reduced NF-κB activity could be associated with inhibition of lung cancer cell growth through the up-regulation of DR3. Our results indicate that the natural toxin BV could be useful as an anti-cancer agent through the overexpression of DR3 and inactivation of NF-κB for the treatment of lung cancer cells and drug resistant cancer cells.

## 4. Experimental Section

### 4.1. Materials

BV was purchased from You-Miel Bee Venom Ltd. (Hwasoon, Jeonnam, Korea). The composition of the BV was as follows: 45%–50% melittin, 2.5%–3% mast cell degranulating peptide, 12% phospholipase A2, 1% lysophospholipase A, 1%–1.5% histidine, 4%–5% 6-pentyl a-pyrone lipids, 0.5% secarpin, 0.1% tertiapin, 0.1% procamine, 1.5%–2% hyaluronidase, 2%–3% amine, 4%–5% carbohydrate, and 19%–27% of others, including protease inhibitor, glucosidase, invertase, acid phosphomonoesterase, dopamine, norepinephrine, and unknown amino acids, with 99.5% purity.

### 4.2. Cell Culture

The human lung cancer cell lines A549 and NCI-H460 as well as normal lung cells LL24 were purchased from the American Type Culture Collection (Manassas, VA, USA). Cell cultures were then maintained at 37 °C in 5% CO_2_ humidified air.

### 4.3. Cell Viability Assay

Cell viability assay was performed as described previously [25].

### 4.4. Apoptosis Evaluation

DAPI and TUNEL staining were performed as described previously [25].

### 4.5. Western Blot Analysis

Western blot analysis was performed as described previously [25]. Membranes were immunoblotted with primary specific antibodies: anti-caspase-3, caspase-9, Bcl-2 (1:1000, Cell Signaling Technology), anti- DR3, DR4, DR5, DR6, TNF-R1, TNF-R2, p50, p65, IκB, p-IκB, Bax and FAS (1/500, Santa Cruz Biotechnology, Dallas, TX, USA) were used in this study.

### 4.6. Transfection (siRNA)

Lung cancer cells (3 × 10^4^ cells/well) were plated in 24-well plates and transiently transfected with siRNA, using a mixture of siRNA and the WelFect-EX PLUS reagent in OPTI-MEN, according to the manufacturer’s specification (WelGENE, Seoul, Korea). The transfected cells were treated with 5 μg/mL of BV for 24 h. DR3 siRNA seq. 5′-GAAGCCCUAAGUACGGUUAtt; DR4 siRNA seq. 5′-CUCUGAUGCUGUUCUUUGAtt; DR6 siRNA seq. 5′-GCCUUCUAGUGUGAUGAAAtt.

### 4.7. Electromobility Shift Assay (EMSA)

Electromobility shift assay was performed as described previously [25].

### 4.8. Immunofluorescence Staining

Immunofluorescence staining was performed as described previously [49]. Primary specific antibodies: anti-p50, p65 (1/500, Santa Cruz Biotechnology, Dallas, TX, USA) were used in this study.

### 4.9. Soft Agar Formation Assay

Cells (8 × 10^3^ per well) were suspended in BME (1 mL with 10% FBS and 0.33% agar) and plated over a layer of solidified BME/10% FBS/0.5% agar (3.5 mL) with BV. Cultures were maintained at 37 °C in a 5% CO_2_ incubator for 10 days, and colonies were counted under a microscope.

### 4.10. Data Analysis

Data were analyzed using the GraphPad Prism 4 ver. 4.03 software (GraphPad Software, La Jolla, CA, USA). Data are presented as mean ± S.D. Differences in all data were assessed by a one-way analysis of variance (ANOVA). Differences were considered significant at *p* < 0.05.

## 5. Conclusions

In this study, we indicated that natural toxin BV could be useful as an anti-cancer agent through the overexpression of DR3 and inactivation of NF-κB for the treatment of lung cancer cells and drug resistant cancer cells. Expression of pro-apoptotic proteins were concomitantly increased, but the NF-κB activity was inhibited. This study suggested that BV induces apoptotic cell death in lung cancer cells through the enhancement of DR3 expression and inhibition of the NF-κB pathway.

## Figures and Tables

**Figure A1:**
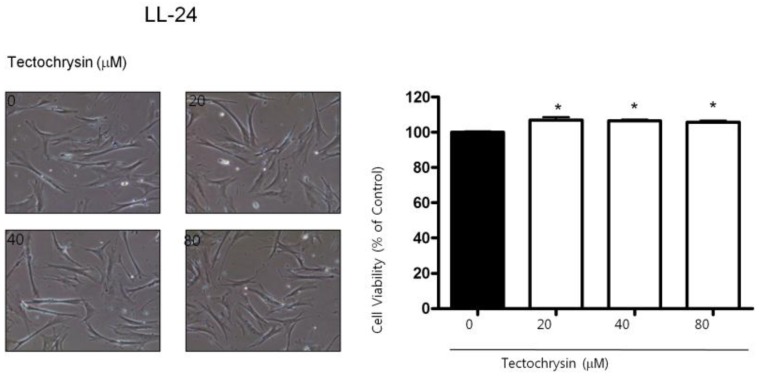
The effect of BV on normal cells.

**Figure A2:**
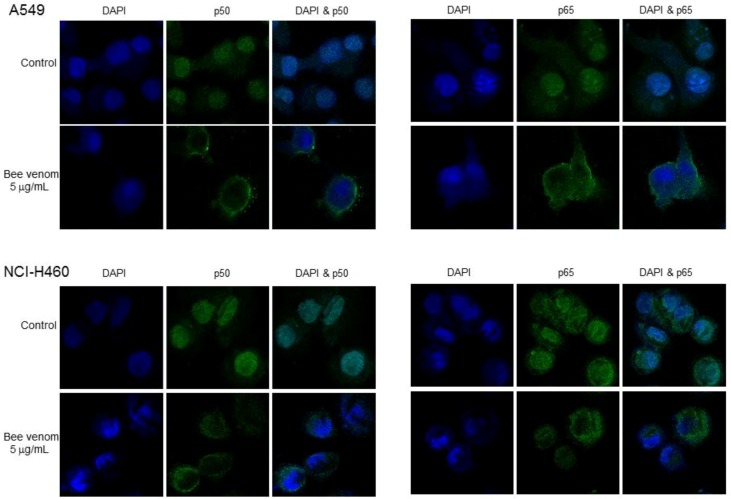
Effect of BV on translocation of p50 and p65 in lung cancer cells. Intracellular location of p50 and p65 in A549 and NCI-H460 cells was determined by an immunefluore-scence confocal scanning microscope (magnification, 400×). Double staining (Merge) with p50 or p65 and DAPI staining demonstrates the localization of p50 and p65 in the nucleus.

## Abbreviations

BV: Bee venom
DRs: Death receptors
EMSA: Electromobility shift assay
FADD: Fas-associated death domain protein
FasL: Fas ligand; FKB: Favokawain B
LLC: Lewis lung carcinoma
MMP-2: Matrix metalloproteinase-2
NF-κB: Nuclear factor kappaB
TNF: Tumor necrosis factor
TRAIL: TNF-related apoptosis-inducing ligand
VEGFR-2: Vascular Endothelial Growth Factor Receptor-2
